# Electrodeposition in Deep Eutectic Solvents: The “Obvious”, the “Unexpected” and the “Wonders”

**DOI:** 10.3390/molecules29143439

**Published:** 2024-07-22

**Authors:** Thomas Doneux, Alassane Sorgho, Fousséni Soma, Quentin Rayée, Moussa Bougouma

**Affiliations:** 1 Chemistry of Surfaces, Interfaces and Nanomaterials (ChemSIN), Faculté des Sciences, Université Libre de Bruxelles (ULB), Boulevard du Triomphe, 2, CP 255, B-1050 Bruxelles, Belgium; 2 Laboratoire de Chimie Analytique, de Physique Spatiale et Energétique (L@CAPSE), UFR/Sciences et Technologies (ST), Université Norbert ZONGO, Avenue Maurice Yameogo, Koudougou BP 376, Burkina Faso

**Keywords:** speciation, apparent pH, reference redox couple, hydrolysis, lability

## Abstract

Deep eutectic solvents (DESs) are attracting considerable attention as non-conventional media for electrodeposition processes. This opinion contribution discusses the debated nature and definition of these solvents as well as some practical considerations of relevance when performing electrodeposition studies in DESs. Using a few illustrative case studies, it is shown that speciation is a key factor determining the electrochemical behaviour of chemical elements in different DESs, and that accounting for the speciation strong similarities can often be found with more conventional or more documented solvents. The need for thermodynamic data is emphasised and it is suggested to expand the composition range of these solvents beyond fixed ratios between the components to exploit the full potentialities of DESs.

## 1. Introduction and Scope

Deep eutectic solvents (DESs) are currently enjoying a surge of popularity in electrochemistry in general and in electrodeposition in particular. Various claimed or established properties explain this popularity, their “greenness” and “low cost” being two of the main driving forces of their attractiveness. At a time when more and more groups are starting to investigate electrodeposition phenomena in DESs, the present contribution aims at sharing some first-hand experience gained during the decade we have been working with such solvents and at discussing some practical considerations that are usually not discussed in research papers. The title and the content of the present paper reflect a presentation delivered by one of us at a meeting of the International Society of Electrochemistry, though in expanded and deepened version. It should be considered an opinion contribution rather than a review. It is by no means comprehensive and has not the ambition of being exhaustive in any of the discussed topics. Readers interested in extensive reviews on DESs would find very valuable information and discussion in thorough reviews by Smith et al. [1], Hansen et al. [2] and Abranches et al. [3], or in the dedicated book by Marcus [4].

Contrary to what the title suggests, the focus of this text is not directly on electrodeposition processes but more on electrochemical considerations of relevance when one considers investigating electrodeposition in DESs. After attempting to introduce the nature of DESs, this text discusses specifically some practical aspects related to electrodeposition in such solvents. Some case studies from the authors’ research are then used to “demystify” these solvents, yet showing some of their genuine scientific interest for fundamental electrodeposition studies.

## 2. What Are Deep Eutectic Solvents?

Introducing a definition is a good entry point to approach the nature of DESs. Here is a first definition:

“A Deep Eutectic Solvent (DES) is a type of ionic liquid formed by combining two or more compounds, typically a hydrogen bond acceptor (HBA) and a hydrogen bond donor (HBD), in specific ratios. DESs are characterized by their eutectic composition, which results in a liquid with a significantly lower melting point than that of the individual components.

These solvents are notable for their unique properties, including: low volatility; high thermal stability; ability to dissolve a wide range of organic and inorganic compounds; tuneable properties; high ionic conductivity; non-toxicity. DESs are often employed as green and sustainable alternatives to traditional solvents in various industrial and scientific applications” [5].

An expert reader will have immediately spotted numerous errors or approximations in this definition, while the non-expert but interested reader would have recognised the generalities read in the introduction section of many publications. This definition was actually adapted from the answers provided by the ChatGPT software. Knowing how such AI applications work, this definition actually reflects a kind of “average” of how DESs are most often defined in the literature, which is a matter of concern in view of its inaccuracy.

DESs are not “a type of ionic liquid”. Ionic liquids are molten salts, i.e., liquids entirely composed of ions, whereas DESs are mixtures that in their liquid state may comprise ions and uncharged entities, or even no ions at all. If it is true that, in many studies, more than two compounds are involved in the mixture, none of the formed mixtures can be qualified as a multicomponent eutectic mixture with reference to an established phase diagram. Actually, in numerous investigations, the invoked DESs are not even eutectic mixtures. Regarding their “unique properties”, the “low” volatility, “high” thermal stability, “non-” toxicity or “high” ionic conductivity are all arguable. Additionally, DESs are not “often” employed in industrial applications.

Turning to scientifically relevant sources, two other definitions are provided here: “DESs are systems formed from a eutectic mixture of Lewis or Brønsted acids and bases which can contain a variety of anionic and/or cationic species” [1]; “Deep eutectic solvents (DESs) are binary mixtures of the definite composition of two components, one of which being ionic, that yield a liquid phase at ambient conditions, ~25 °C” [4]. The first definition is a sentence extracted from a comprehensive review by the group of Abbott, who first developed the DESs and actually coined the term “deep eutectic solvent”. The second definition comes from a book written by Marcus where numerous physico-chemical data about DESs have been compiled. Both descriptions are rather broad, with no specificity related to the “deep” character of the eutectic mixture, but the second is more restrictive since it limits the DESs to two-component systems and imposes the ionic nature of one of the components. This latter restriction is itself arguable because, from a thermodynamic point-of-view, mixtures of non-ionic compounds can be qualified as “deep eutectics” [3]. Those belong to the so-called “Type V” DES [6], in addition to the “Type I” to “Type IV” DESs (in the classification proposed by Abbott [1], Table 1), that indeed all comprise at least one ionic component. Coutinho and colleagues have proposed a definition whereby “deep eutectic solvents are eutectic solvents whose components present significant negative deviations from thermodynamic ideality” [3,7]. If such a definition has the advantage of introducing a sound scientific criterion for the classification and opening the field to non-ionic mixtures, its stringency would de facto exclude many of the currently described Type I-IV DESs, such as the ubiquitous choline chloride–ethylene glycol mixture, which behaves close to ideality from the thermodynamic point of view [8] but is probably the most employed DES in electrodeposition studies. Although this last definition is arguably the most rigorous one, the second definition is probably the one that reflects best what is usually termed a “DES” in the field of electrodeposition, since the ionicity is mandatory. More specifically, it should be recognised that the vast majority of the literature dealing with electrodeposition in DESs actually makes use of the Type III class, i.e., those formed by mixing most often a quaternary ammonium halide (usually choline chloride) with a hydrogen bond donor, i.e., an amide, an alcohol, or a carboxylic acid. Our own expertise with DESs deals with this class, and so the statements and opinions expressed in the present contributions apply to the Type III DESs.

## 3. Practical Aspects in Electrodeposition Investigations

### 3.1. Viscosity

DESs are much more viscous than conventional aqueous and non-aqueous solvents. Among the most popular DESs, the least viscous is ChCl-EG, with *η* ≈ 40 mPa s at 25 °C, while ChCl-U has a value *η* ≈ 800 mPa s at the same temperature [2,4]. Since the viscosity influences the mass transport of electroactive species and the conductivity of the solvent, ill-defined voltammograms are obtained when the viscosity is too high. The presence of water, deliberately added or not, strongly decreases the viscosity, as does an increase in the temperature. While decent voltametric curves can be obtained in ChCl-EG at room temperature, heating is required for most other DESs. Viscosity being thermally activated, it decreases exponentially with temperature so that a moderate elevation of temperature is generally sufficient to perform reliable electrochemical investigations, though ohmic drop issues should not be overlooked [9,10]. In our everyday practice, 60 °C is a good compromise, the viscosity of most DESs being below 100 mPa s at this temperature while avoiding or limiting issues related to the heating of the DESs at higher temperatures.

### 3.2. Thermal Stability and Volatility

As mentioned above in the ChatGPT definition of DESs, a high thermal stability is frequently claimed for DESs. This is definitely incorrect. Under dynamic heating, the onset decomposition temperature can be as low as 100 °C for the ChCl-Malonic acid DES, and only about 10 °C higher for the ubiquitous ChCl-EG [11]. Under isothermal heating for relatively long periods (20 h), mass losses close to 10% are already detected at 50 °C for ChCl-EG [11]. In addition, there is also convincing evidence [12] that esterification reactions take place in DESs prepared from ChCl and a carboxylic acid, processes that are more prominent at higher temperatures but already occurring at room temperature.

If these stability issues are a real concern for genuine industrial applications [13], they do not preclude the pursuit of fundamental research on DESs. In the context of academic research in electrochemistry at the laboratory scale, it is customary practice to work at temperatures around 50–70 °C. At such temperatures, the relatively low volatility of DESs is really an advantage over water or conventional organic solvents, which would undergo significant evaporation in the minute to hours timescale. For the sake of comparison, the vapour pressure at 100 °C has been estimated at 1.3 Pa for ChCl-U and at 11.6 Pa for ChCl-Gly [14], which is much lower than the 101.3 kPa of water but higher than the values reported for ionic liquids.

In our laboratory, experiments have been conducted at 110 °C with ChCl-U [15] or ChCl-Gly [16], without noticing any impact on the investigated electrochemical systems, *on the typical timescale of one-day experiments*. By contrast, in ChCl-Ox (prepared from dihydrated oxalic acid), bubbles already appear in the electrochemical cell at temperatures around 80 °C, which is impractical to conduct experiments, and lower temperatures are thus favoured.

### 3.3. Apparent pH

The rigorous definition of pH and determination of pH scales in DESs are not straightforward. If some considerations employed for non-aqueous solvents are applicable to DESs [17], an additional complexity stems from the fact that DESs are mixtures, and that the nature of their components varies from one DES to the other. Therefore, there would be virtually one pH scale for each DES. This has not deterred researchers from estimating pH of p*K*_a_ values in DESs. In an elegant contribution, Abbott et al. [18], for instance, discussed the concept of Brönsted acidity in such solvents and were able to determine the p*K*_a_ of various carboxylic acids in ChCl-EG and ChCl-Gly. This was achieved by the prior use of an acid–base indicator, bromophenol blue, whose acidity constant was determined by UV-Vis spectrophotometry. In this same contribution, the authors also evaluated the use of conventional glass electrodes to measure the pH in DESs. It was shown that glass electrodes respond reliably to changes in proton activity, though the slope of the potential vs. $-\log a_{H^{+}}$ line departs from the theoretical “Nernstian” value.

The reliability of glass electrodes to probe proton activity in DESs has led many groups to report pH values obtained with conventional pH-meters calibrated against standard aqueous solutions. This very practical approach is commonly found in the literature dealing with non-aqueous solvents, but it is strongly recommended to employ the terminology “apparent pH” instead of “pH” [4] since the measured values do not convey the physical meaning associated with the term “pH”. Table 2 reports some apparent pH values measured for different DESs commonly employed in our laboratory. ChCl-Ox, with a negative apparent pH, behaves as an extremely acidic medium, a property which is shared by many (perhaps all) choline chloride-based DES comprising a carboxylic acid as the hydrogen bond donor. On the contrary, ChCl-U behaves as a slightly basic medium. For ChCl-EG or ChCl-Gly, large variations of apparent pH values are obtained, demonstrating some of the limitations of this practical approach. As shown in Table 2, the measured apparent pH is different depending on the sequence of aqueous buffers effected during the calibration: the displayed value is closer to the pH value of the last (in time) aqueous standard employed. Our interpretation is that ChCl-EG and ChCl-Gly behave as “neutral” DESs in the sense that they have neither a significant acidic nor significant basic character, so that the apparent pH of a solution will be imposed by the solute properties rather than by the DES itself. By contrast, the “acidic” DES ChCl-Ox or the “basic” DES ChCl-U have markedly acidic and basic characters, respectively, that control the apparent pH of the medium. This acido-basic character is sufficiently strong to impose the proton activity at the surface of the glass electrode, independently of the previous calibration sequence, which is not the case of the “neutral” DESs.

### 3.4. Electrochemical Window

**Figure 1:**
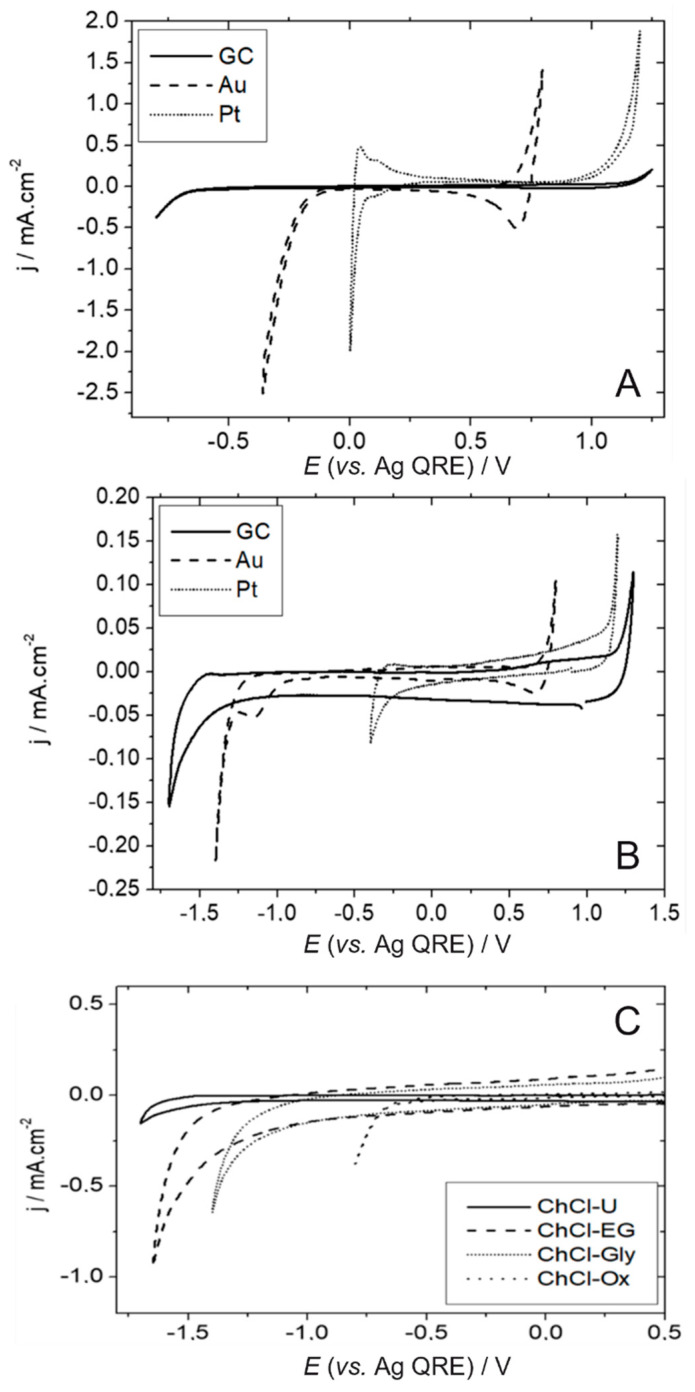
Estimation by cyclic voltammetry of the ideally polarisable domains of different working electrodes (GC, Au, and Pt) in ChCl-Ox (**A**) and ChCl-U (**B**). (**C**) Influence of the nature of the DES on the negative limit of the electrochemical window of a GC electrode. The voltammograms were recorded at a scan rate = 50 mV s^−1^ and at *T* = 60 °C.

A “wide electrochemical window” is often advocated as one of the numerous advantages of DESs over aqueous solvents. Figure 1, which presents cyclic voltammograms recorded at different working electrodes and in different DESs, shows that this is clearly an overstatement. In ChCl-Ox, the width of the ideally polarisable domain (electrochemical window) amounts to ca. 2 V for GC, 1.2 V for Pt and a mere 1 V for Au (Figure 1A). It is slightly more extended in ChCl-U, with values around 3 V for GC, and 1.5 V and 2 V for Au (Figure 1B). For the “acidic” DESs like ChCl-Ox, the negative limit is likely set by the reduction in protons, as attested by the typical reversible signature observed on platinum (reoxidation of H_2_) and the classical sequence of hydrogen evolution reaction activity Pt > Au > GC (Figure 1A). The implication of protons is also likely for other DESs since the limit is the most negative for the “basic” DESs (ChCl-U), then for the “neutral” (ChCl-EG, ChCl-Gly), and then for the “acidic” ones (Figure 1C). At the positive limit, the electrochemical stability of the solvent is bound by the oxidation of chloride anions into chlorine, which is not evolved as gaseous Cl_2_ but solubilised in the Cl_3_^−^ form [19]. Depending on the electrode material, the oxidation of the metal can take place at less positive potentials, as is the case for gold which, owing to the high chloride concentration of the DESs, is oxidised to a soluble gold chloride complex, ${\left[{\mathrm{AuCl}}_{2}\right]}^{-}$ [20,21], explaining the fairly narrow potential window of this material.

### 3.5. Reference Electrodes and Reference Redox Couples

Considering DESs as non-aqueous solvents, the use of conventional aqueous reference electrodes is ill-advised due to the possible contamination of the DES with water and the difficulties in estimating the liquid junction potential. Like in non-aqueous solvents, a common practice consists in using quasi-reference electrodes such as a platinum wire, or silver wire, sometimes previously coated with a layer of AgCl. In our opinion, platinum is a very bad choice, especially if it is directly immersed in the cell. Platinum is the archetype of an inert indicator electrode in potentiometry, because it adjusts its potential to any potential-determining species present in the medium. For this very same reason, it is a bad reference electrode because its potential is prone to drift during the timescale of a typical experimental session. Silver is a much better choice because the potential is determined by the Ag^+^/Ag electrochemical equilibrium, which in turn is dependent on the chloride concentration. Coating the silver wire with AgCl, as is frequently done in aqueous or non-aqueous solvents, is not helpful and can be even counterproductive. Indeed, AgCl is soluble in the chloride-rich DESs in the form of silver chloride complexes such as ${\left[{\mathrm{AgCl}}_{2}\right]}^{-}$, a problem which is also documented in non-aqueous solvents [17]. As a consequence, and this has been plainly demonstrated by Savinell and colleagues [22], the AgCl slowly dissolves in the DESs, resulting in an unstable potential whose value can be dependent on the initial amount of AgCl deposited on the wire. In the same study, the authors showed that by contrast, the potential of a bare silver wire directly immersed in the DES displays stability, an observation that our own practice corroborates. A necessary precaution, though, consists of isolating the silver wire in a tube capped with a porous frit. Immersing a silver wire directly in the main compartment of the electrochemical cell, we realised that metallic silver is solubilised in some DESs such as ChCl-Ox, to the point that during long-time experiments, voltametric peaks associated with the anodic stripping of silver coming from the reference electrode were clearly noticeable on the voltammograms.

**Figure 2:**
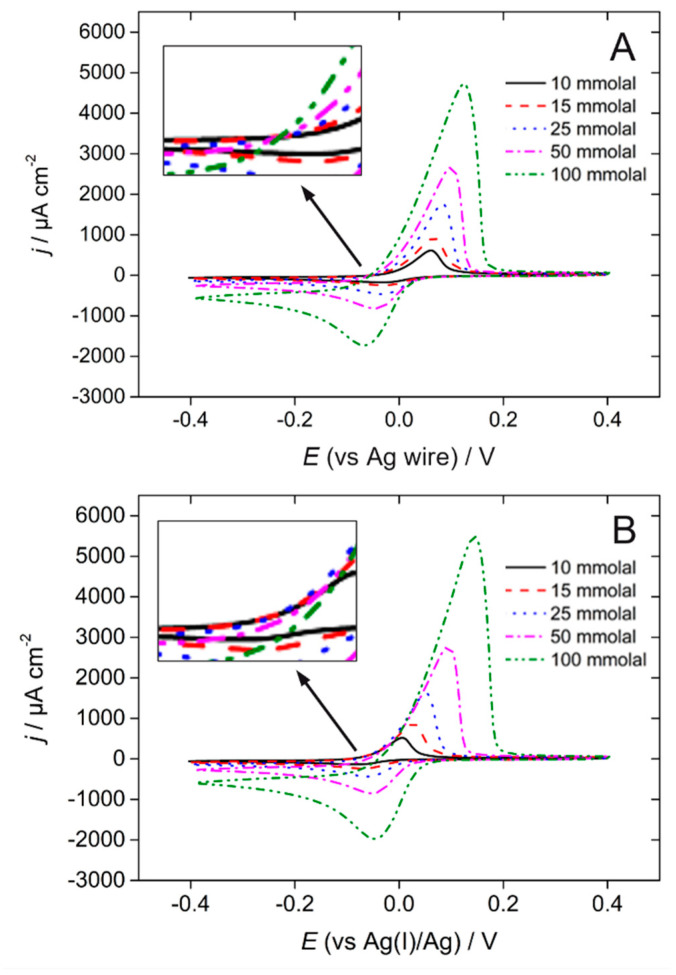
Cyclic voltammograms recorded at an Au electrode in ChCl-U containing different amounts of AgCl as indicated in the legend. In (**A**), the reference electrode was a silver wire directly immersed in the solution. In (**B**) the reference electrode was a silver wire immersed in a ChCl-U + 10 mmolal AgCl solution, separated from the analyte solution by a porous frit. Scan rate = 20 mV s^−1^, *T* = 60 °C. The insets show enlarged parts of the graph around zero current on the positive going sweep.

Besides the experimental requirement of having a reference electrode with a stable and reliable potential in a given DES, identifying one or several suitable reference redox couples to establish potential scales in DESs is of interest for the community. For non-aqueous solvents, the IUPAC recommends the use of either the ferricenium/ferrocene (Fc^+^/Fc) couple or the bis(biphenyl)chromium(I)/(0) (BCr^+^/BCr) couple [23], and it is common practice to add a small amount of one of these species in the electrochemical cell to convert the potentials measured against the employed reference electrode to those expressed in the potential scale of the associated reference redox couple. Such practice is a priori transferable to DESs, though solubility issues make this impractical in some cases. Some authors have employed the [Fe(CN)_6_]^3−/4−^ redox couple as a reference system [24]. Although the Pt|[Fe(CN)_6_]^3−/4−^,ChCl-EG| reference electrode has been demonstrated to be reliable in the ChCl-EG DES [22], the potential of the [Fe(CN)_6_]^3−/4−^ couple in non-aqueous solvents is known to be extremely sensitive to the amount of water [25], which in our opinion makes it unsuitable as a proper reference redox system to draw comparisons between different DESs and under experimental conditions where the amount of water is seldom carefully controlled. By contrast, the Ag^+^/Ag couple is arguably a better choice because its electrochemistry is actually pretty insensitive to the nature of the DES (at least those belonging to Type III comprising choline chloride), exhibiting a similar potential and fast electron transfer kinetics in various DESs [26]. Figure 2 shows two series of voltammograms recorded at gold electrodes in the presence of various amounts of Ag(I) dissolved in ChCl-U [27]. In Figure 2A, a silver wire directly immersed in the electrochemical cell was employed as a reference electrode, whereas in Figure 2B the reference electrode was a silver wire immersed in ChCl-U+10 mmolal AgCl and separated from the cell by a porous frit. All the voltammograms of Figure 2A cross each other at a single point corresponding to a zero current on the positive-going sweep. This is because all the voltammograms are referred to the formal potential of the silver wire (“0” on the potential scale), which is dependent on the concentration of dissolved Ag(I). Importantly, the potential of the crossing point is negative (different from 0), which for an electrodeposition-redissolution phenomenon is indicative of a nucleation-free, fast electron transfer process. In Figure 2B, the zero-current value is shifted in potential between the voltammograms, because the potential of the reference electrode is constant, insensitive to the amount of Ag(I) dissolved in the cell, in contrast to the equilibrium potential of the silver/silver(I) couple measured at the working electrode.

**Figure 3:**
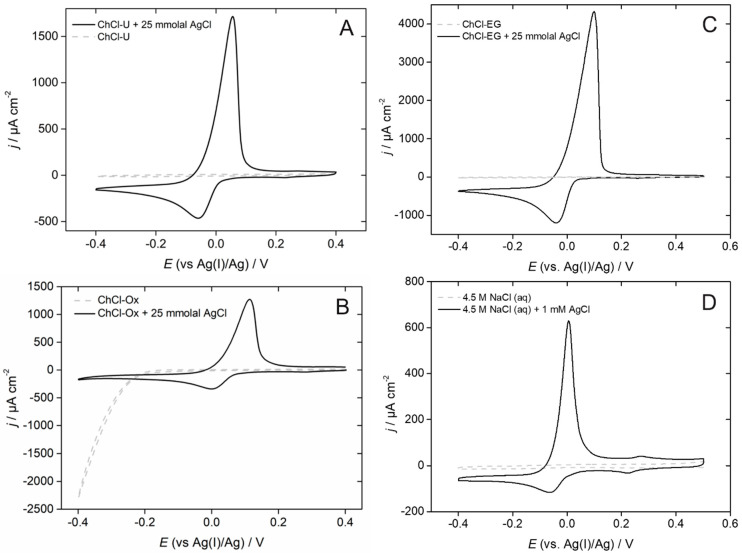
Cyclic voltammograms recorded at Au electrodes in AgCl solutions prepared from ChCl-U (**A**), ChCl-Ox (**B**), ChCl-EG (**C**), and 4.5 M aqueous NaCl. Scan rate = 20 mV s^−1^; *T* = 60 °C (**A**–**C**) or 25 °C (**D**).

When comparing various DESs, the voltametric features associated with the silver electrodeposition-stripping on gold working electrodes are very similar, both in the overpotential and underpotential ranges [26], as illustrated by Figure 3. This is due to an identical speciation of the Ag(I) species in the different DESs, controlled by the high chloride concentration of these media. Incidentally, aqueous solutions of comparably high chloride concentrations also lead to very similar voltammograms [26], as shown in Figure 3D. We thus advocate the use of the Ag^+^/Ag couple both as a practical reference electrode and as a valuable reference redox couple, though a systematic evaluation of the standard potential of this couple in a broad range of DESs would be necessary to substantiate or invalidate this suggestion.

### 3.6. Water Content

The amount of water present in a DES can be (and is often) significant, purposely or not. The presence of water has an impact on many physico-chemical properties, which can be detrimental or beneficial depending on the envisioned application. Some scientists will rightly argue that a DES containing water is a ternary mixture and should thus no longer be considered as a DES. Some others will rightly use the documented evidence that some DESs “retain” their properties such as the hydrogen bonding network up to high water contents. The matter is worth being debated but is closely linked to the very definition of what is a deep eutectic solvent; as explained above, however, there is still no consensus definition of DESs. This should not prevent scientists from exploring the role of water in interesting cases of investigation. In the context of electrodeposition, careful studies have assessed the influence of water on the deposition process, for instance, in the case of nickel electrodeposition [28,29]. Adopting a pragmatic approach, the issue of the water content is connected to the system under investigation and to the purpose of its potential application. DESs are often presented as alternatives to ionic liquids in view of their cost, ease of preparation, or “air-stability”, the latter being indicative that uncontrolled contact between the DES and air is not problematic. If the system of interest is so sensitive to water as to require a glovebox to conduct the experiments, it is likely that a DES is probably not the best choice of solvent. Eliminating water completely from a DES is almost impossible and finely controlling the water content in open-air conditions is extremely tedious. DESs are really advantageous when such considerations can be confidently overlooked. Nevertheless, good laboratory practices should be respected, and some precaution should be taken in preparing and handling the DESs. Considering that choline chloride is a major component of the DESs, ensuring a high level of purity is mandatory. The ChCl salt being hygroscopic, it is often wet upon purchase. It is thus advised to dry it, and in our laboratories, we systematically recrystallise it from ethanol, dry it, and store it in a desiccator prior to use. For the hydrogen bond donor, a case-by-case approach is adopted. Some HBDs are already hydrated, others not; some are more hygroscopic, others less. Sharing a first-hand experience, we have employed in the past anhydrous oxalic acid as the HBD. In addition to being extremely viscous, the prepared DES was prone to readily absorb water from the atmosphere on a minute timescale. As a result, the viscosity was continuously decreasing over time and the intensities of voltametric peaks were evolving accordingly. Purging dry nitrogen into the electrochemical cell proved very efficient in removing water, but to an extent that the viscosity was so high that no decent electrochemical experiment could be performed. On the contrary, when wet nitrogen (pre-saturated with water) was employed to purge the cell, the water content endlessly increased in an uncontrollable way. Nowadays, we tend to employ oxalic acid dihydrate as the HBD for this DES; with 50 mol % of water, it is arguably no longer a DES but it is convenient to employ in a non-controlled atmosphere and enables us to investigate interesting electrochemical systems (vide infra).

## 4. Selected Case Studies

### 4.1. The “Obvious” Copper Electrochemistry

In the previous section, the strong analogies between the electrochemistry of silver in DESs and in chloride-rich aqueous solutions have been highlighted. A similar case can be made with copper. It is known that in *non-complexing* aqueous solutions, Cu(I) is unstable and disproportionate to Cu(0) and Cu(II) because the sequence of standard potentials of the involved redox couples is $E_{\mathrm{Cu}\left(I\right)/\mathrm{Cu}\left(0\right)}^{0}>E_{\mathrm{Cu}\left(\mathrm{II}\right)/\mathrm{Cu}\left(I\right)}^{0}$:

(1) $$\mathrm{Cu}\left(\mathrm{II}\right)\left(\mathrm{aq}\right)+1e^{-}⇌\mathrm{Cu}\left(I\right)\left(\mathrm{aq}\right)\;\;\;E_{\mathrm{Cu}\left(\mathrm{II}\right)/\mathrm{Cu}\left(I\right)}^{0}$$

(2) $$\mathrm{Cu}\left(I\right)\left(\mathrm{aq}\right)+1e^{-}⇌\mathrm{Cu}\left(0\right)\left(s\right)\;\;\;E_{\mathrm{Cu}\left(I\right)/\mathrm{Cu}\left(0\right)}^{0}$$

(3) $$2\mathrm{Cu}\left(I\right)\left(\mathrm{aq}\right)⇌\mathrm{Cu}\left(\mathrm{II}\right)\left(\mathrm{aq}\right)+\mathrm{Cu}\left(0\right)\left(s\right)\;\;\;K_{\mathrm{disp}}=\exp\left[\frac{-2F\left(E_{\mathrm{Cu}\left(\mathrm{II}\right)/\mathrm{Cu}\left(I\right)}^{0}-E_{\mathrm{Cu}\left(I\right)/\mathrm{Cu}\left(0\right)}^{0}\right)}{RT}\right]>1$$

In the presence of *complexing* chloride anions, Cu(I) is comparatively more stabilised than Cu(II), leading to an inversion in the sequence of standard potentials, $E_{\mathrm{Cu}\left(\mathrm{II}\right)/\mathrm{Cu}\left(I\right)}^{0}>E_{\mathrm{Cu}\left(I\right)/\mathrm{Cu}\left(0\right)}^{0}$, and making the comproportionation reaction between Cu(II) and Cu(0) the thermodynamically favourable reaction:

(4) $${\left[{\mathrm{CuCl}}_{x}\right]}^{\left(x-2\right)-}\left(\mathrm{aq}\right)+1e^{-}⇌{\left[{\mathrm{CuCl}}_{y}\right]}^{\left(y-1\right)-}\left(\mathrm{aq}\right)+\left(x-y\right){\mathrm{Cl}}^{-}\left(\mathrm{aq}\right)\;\;\;\;\;\;E_{\mathrm{Cu}\left(\mathrm{II}\right)/\mathrm{Cu}\left(I\right)}^{0}$$

(5) $${\left[{\mathrm{CuCl}}_{y}\right]}^{\left(y-1\right)-}\left(\mathrm{aq}\right)+1e^{-}⇌\mathrm{Cu}\left(0\right)\left(s\right)+{\mathrm{yCl}}^{-}\left(\mathrm{aq}\right)\;\;\;\;\;\;E_{\mathrm{Cu}\left(I\right)/\mathrm{Cu}\left(0\right)}^{0}$$

$$2{\left[{\mathrm{CuCl}}_{y}\right]}^{\left(y-1\right)-}\left(\mathrm{aq}\right)+\left(x-y\right){\mathrm{Cl}}^{-}\left(\mathrm{aq}\right)⇌{\left[{\mathrm{CuCl}}_{x}\right]}^{\left(x-2\right)-}\left(\mathrm{aq}\right)+\mathrm{Cu}\left(0\right)\left(s\right)$$

(6) $$K_{\mathrm{disp}}=\exp\left[\frac{-2F\left(E_{\mathrm{Cu}\left(\mathrm{II}\right)/\mathrm{Cu}\left(I\right)}^{0}-E_{\mathrm{Cu}\left(I\right)/\mathrm{Cu}\left(0\right)}^{0}\right)}{RT}\right]<1$$

**Figure 4:**
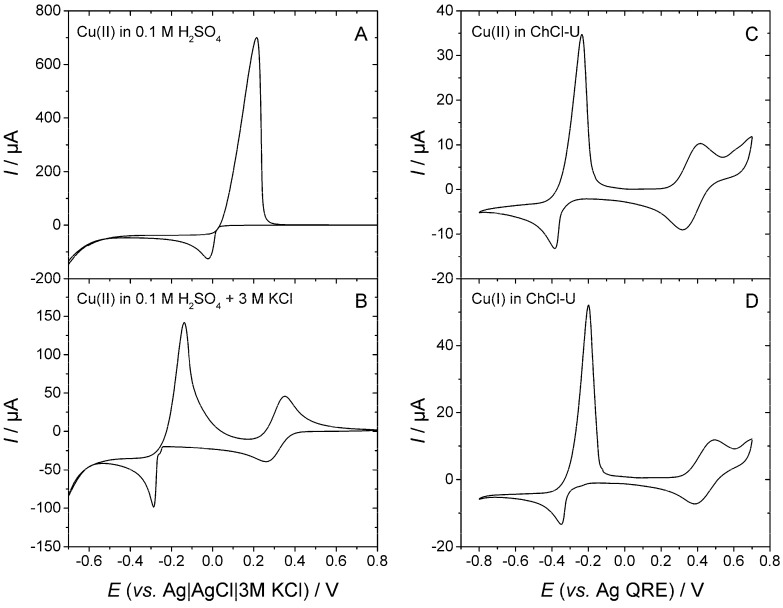
Voltametric behaviour of copper recorded in different media: (**A**) 10 mM CuSO_4_ in aqueous 0.1 M H_2_SO_4_, scan rate = 10 mV s^−1^; *T* = 25 °C; glassy carbon working electrode (0.0707 cm^2^); (**B**) 10 mM CuSO_4_ in aqueous 0.1 M H_2_SO_4_ + 3 M KCl, scan rate = 10 mV s^−1^; *T* = 25 °C; glassy carbon working electrode (0.0707 cm^2^); (**C**) 10 mmolal CuO in ChCl-U, scan rate = 20 mV s^−1^; *T* = 110 °C; gold working electrode (0.0201 cm^2^); (**D**) 10 mmolal Cu_2_O in ChCl-U, scan rate = 20 mV s^−1^; *T* = 110 °C; gold working electrode (0.0201 cm^2^).

Figure 4 shows cyclic voltammograms recorded in aqueous or DES solutions of copper precursors. In a non-complexing aqueous solution of Cu^2+^ (Figure 4A), only one pair of peaks is observed, corresponding to the 2e^−^ reduction of Cu(II) to Cu(0) for the cathodic peak and the re-oxidation of Cu(0) to Cu(II) for the anodic peak. Upon addition of a large amount of chloride (Figure 4B), the voltammogram displays two pairs of peaks assigned to the Cu(II)/Cu(I) and Cu(I)/Cu(0) couples for the peaks at the most positive and most negative potentials, respectively, in agreement with the thermodynamic considerations exposed above. In the ChCl-U DES, the voltammogram recorded in the presence of either Cu(II) (Figure 4C) or Cu(I) (Figure 4D) dissolved in solution is reminiscent of that obtained in aqueous, chloride-rich solutions, with two well-separated pairs of peaks again associated with the Cu(II)/Cu(I) and Cu(I)/Cu(0) couples [30,31].

Such a voltametric behaviour has been documented in other DESs, such as ChCl-EG [32,33,34,35] or ChCl-trifluoroacetamide [36], and it has been shown that this morphology is preserved after addition of up to 95% of water (in volume fraction, in the ChCl-EG DES) [33,34]. As impressive as it might sound, this fact does not stem from any peculiar characteristic of the DES but simply from its high concentration of chloride; for the sake of comparison, the mole fraction of water in the aqueous solution employed for Figure 4B is ~95%, yet two pairs of peaks are clearly discerned. The electrochemical behaviour of copper in DES is thus essentially controlled by the speciation of the Cu(I) and Cu(II) species, which is actually rather similar to that of aqueous, chloride-rich, solutions. In both cases, the same cuprous species ${\left[{\mathrm{CuCl}}_{y}\right]}^{\left(y-1\right)-}$ and cupric species ${\left[{\mathrm{CuCl}}_{x}\right]}^{\left(x-2\right)-}$ have been identified as the main entities present in solution [35,37,38]. Nevertheless, the actual distribution between the different chloro complexes depends on the temperature and on the composition of the solution (e.g., concentration of chloride) and can thus vary between different DESs. In contrast with aqueous media for which numerous equilibrium constants (solubility products, stability constants, dissociation constants, etc.) have been determined, thermodynamic data are extremely scarce in DESs, which is detrimental to the scientific development of field.

It is interesting to remark that even though copper is present as a mixture of different chloro complexes in DESs, only one pair of peaks associated with the Cu(II)/Cu(I) and Cu(I)/Cu(0) couples are observed in the voltammograms. This indicates that the interconversion between the different copper chloro complexes is fast in comparison to the voltametric timescale, indicating that chloride ligands are rather labile. If the complexes were inert, the interconversion would be slow (compared to the voltametric timescale) and different voltametric peaks would be observed, whose relative intensities would reflect the proportions between the different complexes.

A very similar discussion applies to the case of silver, for which various chloro complexes have been identified in DESs [37], and which also displays only one voltametric peak for the Ag(I)/Ag(0) couple [39].

### 4.2. The “Unexpected” Tellurium Electrochemistry

**Figure 5:**
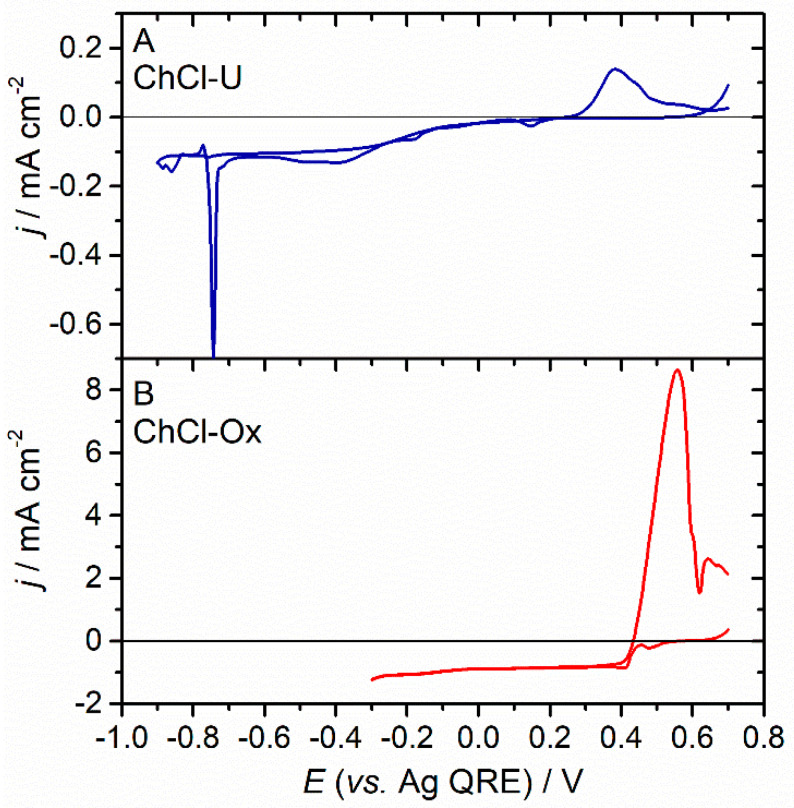
Voltametric behaviour of Te(IV) recorded at a rotating disc Au electrode in ChCl-U + 10 mmolal TeCl_4_ (**A**) or ChCl-Ox + 10 mmolal TeCl_4_ (**B**). Scan rate = 10 mV s^−1^; *T* = 70 °C; rotation rate 1500 rpm.

At this stage, it is tempting to consider that the electrochemistry of a given element in any choline chloride-based DES would be very similar to the electrochemistry of this same element in a chloride-rich aqueous solution. This statement is contradicted by the case of tellurium, which we have recently investigated [40]. Figure 5 shows the voltammograms recorded at a gold rotating disc electrode for a solution of Te(IV) dissolved in the ChCl-U or ChCl-Ox DES. In sharp contrast to the cases of silver or copper for which the voltametric features were similar regardless of the nature of the DES, very different voltammograms are obtained in the present case. This behaviour can be again interpreted in terms of speciation, though it is not solely controlled by the high concentration of chloride but also by the apparent pH of the medium. As mentioned previously, ChCl-U is slightly “basic”, whereas ChCl-Ox is extremely “acidic”. In aqueous solutions, tellurium(IV) chloro complexes are known to be prone to hydrolysis, chloride ligands being progressively replaced by hydroxide ligands as the pH increases [41,42]. The chloro complex ${\left[{\mathrm{TeCl}}_{6}\right]}^{2-}$ is the predominant Te(IV) species in ChCl-Ox but is completely absent in ChCl-U, where the dominant species are likely mixed hydroxo-chloro complexes [40]. Additionally, Te(IV) chloro complexes are labile, whereas hydroxo-chloro complexes are inert. This is reflected in the voltametric characteristics, with very distinct potentials and electrode kinetics being associated with the different peaks observed in ChCl-U or ChCl-Ox.

Considering the reduction of Te(IV) to Te(0), the global reaction can be actually decomposed in at least two steps, a chemical reaction and an electron transfer reaction. Equations (7)–(9) illustrate the case of ChCl-Ox:

(7) $${\left[{\mathrm{TeCl}}_{6}\right]}^{2-}+4e^{-}⇌\mathrm{Te}\left(s\right)+6{\mathrm{Cl}}^{-}\;\;\;\mathrm{global},\;\mathrm{fast}$$

(8) $${\left[{\mathrm{TeCl}}_{6}\right]}^{2-}\begin{array}{c} \vec{k}\\ ⇌\\ \overset{↼}{k} \end{array}\mathrm{Te}\left(\mathrm{IV}\right)+6{\mathrm{Cl}}^{-}\;\;\;\mathrm{chemical},\;\mathrm{fast}$$

(9) $$\mathrm{Te}\left(\mathrm{IV}\right)+4e^{-}\begin{array}{c} k^{0}\\ ⇌ \end{array}\mathrm{Te}\left(s\right)\;\;\;\;\;\;\mathrm{electron}\;\mathrm{transfer},\;\mathrm{fast}$$

and Equations (10)–(12) that of ChCl-U:

(10) $${\left[\mathrm{Te}{\left(\mathrm{OH}\right)}_{x}{\mathrm{Cl}}_{y}\right]}^{\left(x+y-4\right)-}+4e^{-}⇌\mathrm{Te}\left(s\right)+{\mathrm{yCl}}^{-}+{\mathrm{xOH}}^{-}\;\;\;\mathrm{global},\;\mathrm{slow}$$

(11) $${\left[\mathrm{Te}{\left(\mathrm{OH}\right)}_{x}{\mathrm{Cl}}_{y}\right]}^{\left(x+y-4\right)-}\begin{array}{c} \vec{k}\\ ⇌\\ \overset{↼}{k} \end{array}\mathrm{Te}\left(\mathrm{IV}\right)+{\mathrm{yCl}}^{-}+{\mathrm{xOH}}^{-}\;\;\;\;\;\;\mathrm{chemical},\;\mathrm{slow}$$

(12) $$\mathrm{Te}\left(\mathrm{IV}\right)+4e^{-}\begin{array}{c} k^{0}\\ ⇌ \end{array}\mathrm{Te}\left(s\right)\;\;\;\;\;\;\mathrm{electron}\;\mathrm{transfer},\;\mathrm{fast}$$

For the global reaction (7) to appear electrochemically reversible, i.e., fast as compared to the voltametric timescale, both the chemical reactions (8) and the electron transfer reaction (9) must be fast as well. Consequently, the fact that in ChCl-U the global reaction (10) is electrochemically slow implies that the chemical reaction (11) is kinetically limiting, pointing to the inertness of the mixed hydroxo-chloro complexes.

In the present case of Te(IV), the speciation is not only determined by the high concentration of chloride anions in the solvent, but also by the “acidity” of the solvent. It is enlightening to realise that while the Te(IV) precursor TeCl_4_ is soluble in both the “acidic” ChCl-Ox and the “basic” ChCl-U, the precursor TeO_2_ is soluble in the former but not in the latter. Interestingly, when TeCl_4_ is dissolved in the “neutral” ChCl-EG, some apparent discrepancies between the voltametric behaviours have been reported by different authors. Perry et al. [43] obtained voltammograms with electrochemically reversible peaks analogous to those recorded in ChCl-Ox, but dos Santos et al. [44,45] obtained much less reversible waves, closer to the responses seen in the ChCl-U DES. As discussed in a previous section, we postulate that owing to the extremely weak acido-basic character of the “neutral” ChCl-EG solvent, the apparent pH is imposed by the solute and not by the solvent. Because TeCl_4_ has a rather strong Lewis acidity, it is likely that the resulting solution has a pretty low apparent pH (though no values were mentioned in the publications) and that this value is quite sensitive to the exact preparation conditions of the solution (Te(IV) concentration, purity of chemicals, traces of water, preparation of the DES mixture, temperature, etc.).

Although we are not aware of any comparable behaviour reported in the literature, it is likely that similarities can be expected with other chlorometallates prone to hydrolysis.

## 5. Conclusions and Perspectives on the “Wonders” of DESs

- There is currently no consensus definition of what a DES is. In the words of colleagues, “common parlance has adopted the term “deep eutectic solvent” […] as the catch-all term for [a] diverse class of materials” [2], and a close examination reveals that it is often a misnomer. For the scientific edification of the field, the emergence of a clear and consensual definition is necessary, though at the “risk” that some or even many of the mixtures that are nowadays called “DESs” would not fit in such definition.

- Superlatives are frequently found in the literature dealing with DESs, which are presented with *wonderful* properties. However, as this contribution shows, a lot of it should be “demystified”, as many observations can be easily rationalised on the grounds of well-established chemistries. In our own investigations, experience has taught us that for every electrochemical system that we have investigated, close analogies could be found (to some extent) in the existing literature regarding similar systems in other solvents. The case studies presented above illustrated that looking at chloride-rich aqueous solvents and taking into account pH effects can satisfactorily help explaining experimental observations made in DESs. In various instances, not discussed here, we have noticed similarities between the electrochemical behaviours recorded in DESs and those recorded in chloride-containing ionic liquids or molten salts, and frequently with those obtained in room temperature haloaluminate ionic liquids. Interestingly, these latter solvents historically found potentialities in the electrochemical processing of transition metal halides prone to hydrolysis [46]. We estimate that emphasising the analogies (and singularities) with other solvents could be beneficial for an improved understanding of these solvents, as well as other “non-conventional” solvents. In some aspects, DESs bear similarities with water-in-salt-electrolytes, with ionic liquids, and with concentrated brines [47], so identifying connections and highlighting dissimilarities between them would deepen our molecular understanding of these media.

- Even though DESs are ill-defined and the electrochemistry of various elements are not specific to the employed DES, these solvents deserve to be investigated further because they exhibit interesting electrochemistries that are indeed sensitive to the exact nature of the DES. If the real potentialities of DESs for industrial applications is questionable [13], the intrinsic modularity of DESs offers many exciting prospects for fundamental research.

Chalcogen electrochemistries (tellurium, selenium) are in our opinion good illustrations. It is somehow remarkable that the electrochemistry of a species such as ${\left[{\mathrm{TeCl}}_{6}\right]}^{2-}$ can be readily conducted in the water-loaded ChCl-Ox DES, whereas in aqueous medium it would require the use of a >7 M HCl solution; in haloaluminate ionic liquids, the use of a glove box would be mandatory because of the very strong sensitivity of the solvent to water; and in molten salts, much higher temperatures would be needed. From a practical point-of-view the possibility of working in the 50–110 °C range is a decent advantage over water and conventional organic solvents, for which significant evaporation would require close management. For example, a temperature of 80 °C was found ideal for the growth of tellurium nanowires in ChCl-EG [43], and selenium is known to crystallise in the conductive trigonal form only above ~70 °C. Taking advantage of the (relative) stability of DESs at such temperatures, we have been able to investigate the electrochemical formation of selenium and of binary metal–selenium compounds [15,16,31,48].

**Scheme 1:**
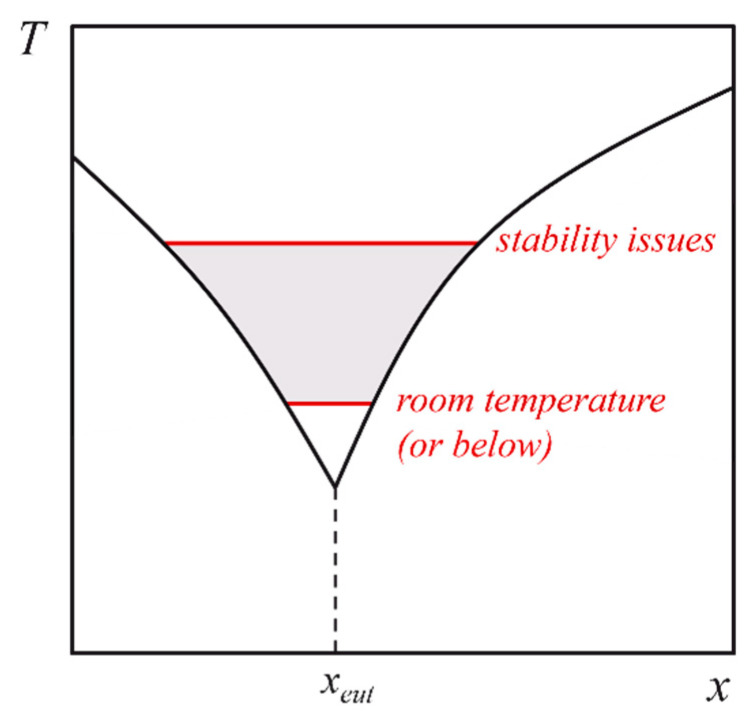
Phase diagram of a putative DES. The shaded region is delimited by the solidification lines (in black) and by the highest and lowest practical temperatures (in red).

- Although more and more data about the physico-chemical properties of DESs are reported, the availability of thermodynamic data such as acidity constants, solubility products, formation constants, or standard potentials is extremely scarce. This type of information would be extremely useful for electrodeposition investigations and, conversely, electrodeposition studies can contribute to gathering these data directly or indirectly (for instance by estimating a chemical constant from a shift of peak potential).

- In line with other authors [2,3], we find opportunities in expanding the composition range of DESs. While the adjective “eutectic” involves a specific composition of the mixture, the interest in DESs stems from their properties, and some of those are unrelated to the eutectic proportions between the components of the mixture. As in the case of the aforementioned room temperature haloaluminate ionic liquids, which are classified as “basic” or “acidic” depending on the ratio between the organic salt and the aluminium halide, it is well possible that the “acidity” (or another property) of a DES could be modulated through its composition beyond the eutectic one, giving an additional degree of freedom in the versatility of these solvents. We thus advocate the exploration of the entire trapezoid shaded region of the phase diagram (see Scheme 1) in terms of composition and temperature range.

## Figures and Tables

**Table 1:** Classification of ionic DESs ^a^.

Class	Composition ^b^
Type I	Cat^+^X^−^zMCl_x_
Type II	Cat^+^X^−^zMCl_x_·yH_2_O
Type III	Cat^+^X^−^zRZ
Type IV	${\mathrm{MCl}}_{x}+\mathrm{RZ}\to {\mathrm{MCl}}_{x-1}^{+}\cdot \mathrm{RZ}+{\mathrm{MCl}}_{x+1}^{-}$

^a^ according to the classification proposed by Abbott and colleagues [1]. ^b^ Cat^+^ is an ammonium, a phosphonium, or a sulfonium cation (most often a quaternary ammonium, a pyridinium or an imidazolium); X^−^ is a halide anion (most often Cl^−^); MCl_x_ is a metal chloride salt; RZ is an organic amide (e.g., urea), a carboxylic acid (e.g., oxalic acid) or an alcohol (e.g., ethylene glycol).

**Table 2:** Apparent pH of selected DESs at 60 °C.

Deep Eutectic Solvent	Apparent pH ^a^
ChCl-Ox	−1.1
ChCl-EG	6.0 ^b^; 8.4 ^c^; 9.8 ^d^
ChCl-Gly	4.5 ^b^; 8.2 ^c^; 8.7 ^d^
ChCl-U	8.1

^a^ Readings at 60 °C of a glass electrode pH-meter previously calibrated with aqueous standard buffer solutions at room temperature. ^b^ After last calibration point in buffer pH 4.0. ^c^ After last calibration point in buffer pH 7.0. ^d^ After last calibration point in buffer pH 10.0.
